# *N*-[(2-Hydr­oxy-1-naphth­yl)(2-hydroxy­phen­yl)meth­yl]acetamide

**DOI:** 10.1107/S1600536809007983

**Published:** 2009-03-11

**Authors:** M. NizamMohideen, S. Thenmozhi, A. SubbiahPandi, N. Panneer Selvam, P. T. Perumal

**Affiliations:** a Department of Physics, The New College (Autonomous), Chennai 600 014, India; b Department of Physics, Presidency College (Autonomous), Chennai 600 005, India; c Organic Chemistry Division, Central Leather Research Institute, Chennai 600 020, India

## Abstract

In the asymmetric unit of the title compound, C_19_H_17_NO_3_, there are two crystallographically independent mol­ecules, which are connected to each other by O—H⋯O hydrogen bonds, forming mol­ecular chains as well as cyclic centrosymmetric *R*_2_^2^(16) dimers.

## Related literature

For background literature, see: Barker *et al.* (2008 ▶); Gade (2002 ▶); Linton & Hamilton (1997 ▶); Valeur & Leray (2000 ▶); Wabnitz & Spencer (2002 ▶). For related structures, see: Gowda *et al.* (2000 ▶, 2006 ▶, 2007 ▶). For hydrogen-bond motifs, see: Bernstein *et al.* (1995 ▶). For bond-length data, see: Fun *et al.* (2008 ▶).
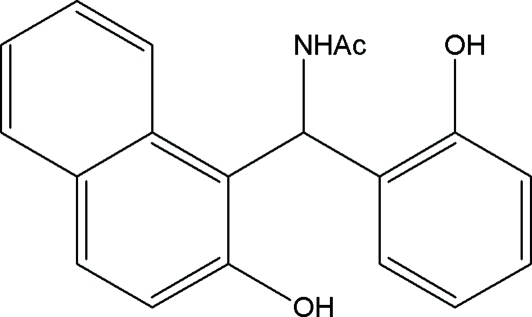


## Experimental

### 

#### Crystal data

C_19_H_17_NO_3_
*M* *_r_* = 307.34
Monoclinic, 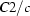

*a* = 21.286 (5) Å
*b* = 17.9288 (4) Å
*c* = 19.524 (7) Å
β = 121.428 (1)°
*V* = 6358 (3) Å^3^
*Z* = 16
Mo *K*α radiation
μ = 0.09 mm^−1^
*T* = 293 K
0.20 × 0.16 × 0.16 mm

#### Data collection

Bruker Kappa APEXII CCD diffractometer
Absorption correction: multi-scan (*SADABS*; Bruker, 2004 ▶) *T*_min_ = 0.980, *T*_max_ = 0.986
33278 measured reflections
6464 independent reflections
4384 reflections with *I* > 2σ(*I*)
*R*_int_ = 0.034

#### Refinement

*R*[*F* ^2^ > 2σ(*F* ^2^)] = 0.045
*wR*(*F* ^2^) = 0.146
*S* = 1.02
6464 reflections
417 parameters
H-atom parameters constrained
Δρ_max_ = 0.47 e Å^−3^
Δρ_min_ = −0.50 e Å^−3^

### 

Data collection: *APEX2* (Bruker, 2004 ▶); cell refinement: *APEX2* and *SAINT* (Bruker, 2004 ▶); data reduction: *SAINT* and *XPREP* (Bruker, 2004 ▶); program(s) used to solve structure: *SHELXS97* (Sheldrick, 2008 ▶); program(s) used to refine structure: *SHELXL97* (Sheldrick, 2008 ▶); molecular graphics: *ORTEP-3 for Windows* (Farrugia, 1997 ▶); software used to prepare material for publication: *SHELXL97* and *PLATON* (Spek, 2009 ▶).

## Supplementary Material

Crystal structure: contains datablocks global, I. DOI: 10.1107/S1600536809007983/pv2141sup1.cif

Structure factors: contains datablocks I. DOI: 10.1107/S1600536809007983/pv2141Isup2.hkl

Additional supplementary materials:  crystallographic information; 3D view; checkCIF report

## Figures and Tables

**Table 1:** Hydrogen-bond geometry (Å, °)

*D*—H⋯*A*	*D*—H	H⋯*A*	*D*⋯*A*	*D*—H⋯*A*
O1—H1⋯O3^i^	0.82	1.99	2.792 (2)	166
O2*A*—H2*A*1⋯O3^ii^	0.82	1.89	2.702 (2)	170
O2—H2⋯O3*A*^iii^	0.82	1.81	2.588 (2)	159
O1*A*—H1*A*1⋯O3*A*	0.82	2.16	2.928 (2)	156

Symmetry codes: (i) 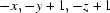; (ii) 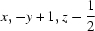; (iii) 
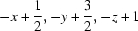
.

**Fig. 1.**
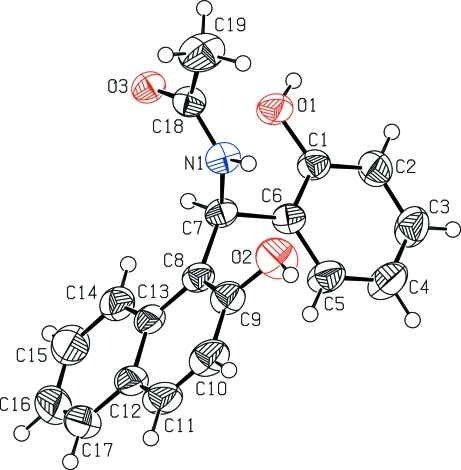
One of the two independent molecules in the asymmetric unit of (I), showing the atom-numbering scheme. Displacement ellipsoids are drawn at the 30% probability level.

**Fig. 2.**
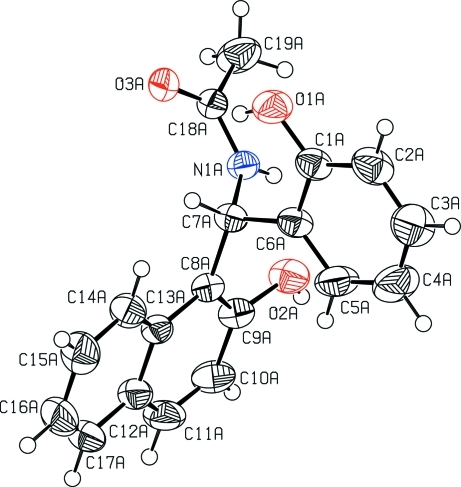
The other independent molecules in the asymmetric unit of (I), showing the atom-numbering scheme. Displacement ellipsoids are drawn at the 30% probability level.

**Fig. 3.**
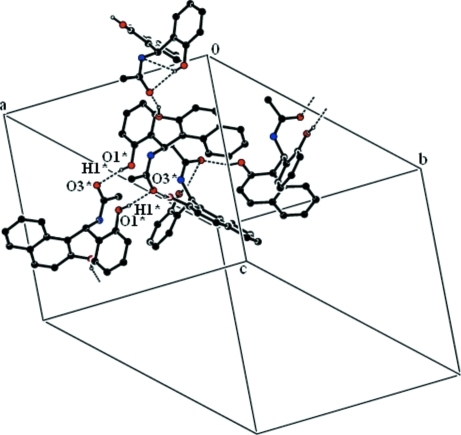
Part of the crystal structure of (I), showing the R22(16) rings. For the sake of clarity, H atoms not involved in the hydrogen bonding have been omitted for clarity. Hydrogen bonding is shown as dashed lines. [Symmetry codes: (*) -x, -y + 1, -z + 1]

## supplementary crystallographic information

### Comment

β-Acetamido ketones serve as potential intermediates in the synthesis of natural products and antibiotics (Wabnitz & Spencer, 2002). Due to the nucleophilic nature of benzylic hydroxyl groups these are usually protected during multi-step organic synthesis (Barker *et al.*, 2008). Amide moiety and their metal ion complexes are widely used for their properties and potential applications (Gade, 2002; Valeur & Leray, 2000; Linton & Hamilton, 1997). The amide linkage [–NHC(O)-] is known to be strong enough to form and maintain protein architectures and has been utilized to create various molecular devices for a spectrum of purposes in organic chemistry. The effect of substituents on the solid state structures of N-aromatic amides have been described in the literature (Gowda *et al.*, 2000, 2006, 2007). As part of our investigations on acetamide derivatives, the title compound, (I), has been prepared and its crystal structure is presented here.

Figs. 1 and 2 show the molecular structures and conformations of the two crystallographically independent molecules, A (C1—C19, N1, O1, O2, O3) and B (C1A—C19A, N1A, O1A, O2A, O3A), in the asymmetric unit of (I), with the atomic numbering scheme. The bond lengths and angles in the two independent molecules agree with each other. The normal probability plot analyses (International Tables for X-ray Crystallography, 1974, Vol. IV, pp. 293–309) for both bond lengths and angles show that the differences between the two symmetry independent molecules are of a statistical nature. The bond distances of C18 = O3 and C18A = O3A [1.245 (2) and 1.244 (2) Å] for the molecules A and B, respectively, which are typical for double bonds (Fun *et al.*, 2008).

In the molecules A and B, benzene and naphthalene rings are individually planar as expected. The deviations of the atoms O2 and O2A from the least-squares plane of the naphthalene rings are -0.075 (1) and 0.164 (1) Å. The deviations of the atoms O1 and O1A from the least-squares plane of the benzene rings are 0.056 (1) and 0.021 (1) Å. The dihedral angles between the naphthalene ring system and benzene rings are 75.7 (1) and 82.9 (1)° for molecules A and B respectively, and those between the fused rings are 0.3 (1) and 2.8 (1) °.

The crystal packing is stabilized by strong O—H···O inter and intramolecular hydrogen bonds and each molecule has a week intramolecular C—H···O interaction (Table 1). Considering only A-type molecules, atom O1 acts as a donar in a strong intermolecular O—H···O interaction *via* H1 with acetamido atom O3 of a symmetry related molecule, generating centrosymmetric hydrogen bonded dimers with a cyclic *R*_2_^2^(16) ring system (Bernstein *et al.*, 1995) (Fig. 3). The interlinking of A and B molecules *via* strong O—H···O hydrogen bond generates infinite chains running along *c* axis. The atoms O3 and O3a act as a acceptors for all inter and intramolecular interactions.

### Experimental

A mixture of 2-hydroxybenzaldehyde (10 mmol), β-naphthol (10 mmol) and iodine (0.4 mmol, 4 mol%) were mixed in acetonitrile (5 ml). To the suspension acetyl chloride (2.8 mmol, 0.2 ml) was added and the reaction mixture was stirred at room temperature for 6 h. After the completion of the reaction (as monitored by TLC), saturated sodium thiosulfate solution (5 ml) was added. The precipitated solid was filtered and dried. The dried sample was washed with diethyl ether (2 × 10 ml) and again dried. Single crystals of the title compound suitable for X-ray diffraction were obtained by slow evaporation of a solution in ethanol.

### Refinement

All H atoms were positioned geometrically, with N—H = 0.86, O—H = 0.82 and C—H = 0.93, 0.98 and 0.96 Å, for aromatic, methylene and methyl H-atoms, respectively, and constrained to ride on their parent atoms, with *U*_iso_(H) = *xU*_eq_(C, N), where *x* = 1.5 for methyl H, and *x* = 1.2 for all other H atoms.

### Crystal data

**Table:**

C_19_H_17_NO_3_	*F*(000) = 2592
*M**_r_* = 307.34	*D*_x_ = 1.284 Mg m^−^^3^
Monoclinic, *C*2/*c*	Mo *K*α radiation, λ = 0.71073 Å
Hall symbol: -C 2yc	Cell parameters from 6464 reflections
*a* = 21.286 (5) Å	θ = 2.5–25°
*b* = 17.9288 (4) Å	µ = 0.09 mm^−^^1^
*c* = 19.524 (7) Å	*T* = 293 K
β = 121.428 (1)°	Prism, colourless
*V* = 6358 (3) Å^3^	0.20 × 0.16 × 0.16 mm
*Z* = 16	

### Data collection

**Table:**

Bruker Kappa APEXII CCD diffractometer	6464 independent reflections
Radiation source: fine-focus sealed tube	4384 reflections with *I* > 2σ(*I*)
graphite	*R*_int_ = 0.034
ω and φ scans	θ_max_ = 26.4°, θ_min_ = 2.2°
Absorption correction: multi-scan (*SADABS*; Bruker, 2004)	*h* = −26→26
*T*_min_ = 0.980, *T*_max_ = 0.986	*k* = −22→22
33278 measured reflections	*l* = −24→23

### Refinement

**Table:**

Refinement on *F*^2^	Primary atom site location: structure-invariant direct methods
Least-squares matrix: full	Secondary atom site location: difference Fourier map
*R*[*F*^2^ > 2σ(*F*^2^)] = 0.045	Hydrogen site location: inferred from neighbouring sites
*wR*(*F*^2^) = 0.146	H-atom parameters constrained
*S* = 1.02	*w* = 1/[σ^2^(*F*_o_^2^) + (0.0747*P*)^2^ + 2.6626*P*] where *P* = (*F*_o_^2^ + 2*F*_c_^2^)/3
6464 reflections	(Δ/σ)_max_ = 0.005
417 parameters	Δρ_max_ = 0.47 e Å^−^^3^
0 restraints	Δρ_min_ = −0.49 e Å^−^^3^

### Special details

**Table:**

Geometry. All e.s.d.'s (except the e.s.d. in the dihedral angle between two l.s. planes) are estimated using the full covariance matrix. The cell e.s.d.'s are taken into account individually in the estimation of e.s.d.'s in distances, angles and torsion angles; correlations between e.s.d.'s in cell parameters are only used when they are defined by crystal symmetry. An approximate (isotropic) treatment of cell e.s.d.'s is used for estimating e.s.d.'s involving l.s. planes.
Refinement. Refinement of *F*^2^ against ALL reflections. The weighted *R*-factor *wR* and goodness of fit *S* are based on *F*^2^, conventional *R*-factors *R* are based on *F*, with *F* set to zero for negative *F*^2^. The threshold expression of *F*^2^ > σ(*F*^2^) is used only for calculating *R*-factors(gt) *etc*. and is not relevant to the choice of reflections for refinement. *R*-factors based on *F*^2^ are statistically about twice as large as those based on *F*, and *R*- factors based on ALL data will be even larger.

### Fractional atomic coordinates and isotropic or equivalent isotropic displacement parameters (Å^2^)

**Table:**

	*x*	*y*	*z*	*U*_iso_*/*U*_eq_	
O1	0.05068 (7)	0.50195 (8)	0.59220 (8)	0.0556 (4)	
H1	0.0080	0.5100	0.5793	0.083*	
O2	0.30227 (7)	0.59048 (8)	0.70861 (10)	0.0655 (4)	
H2	0.3361	0.6207	0.7274	0.098*	
O3	0.09894 (7)	0.46622 (7)	0.47788 (7)	0.0427 (3)	
N1	0.17949 (8)	0.53425 (8)	0.58664 (9)	0.0413 (4)	
H1A	0.2011	0.5768	0.6017	0.050*	
C1	0.09638 (10)	0.51566 (10)	0.67223 (11)	0.0415 (4)	
C2	0.07009 (11)	0.53887 (11)	0.72002 (13)	0.0531 (5)	
H2A	0.0200	0.5475	0.6975	0.064*	
C3	0.11779 (12)	0.54928 (13)	0.80092 (14)	0.0619 (6)	
H3	0.1000	0.5648	0.8332	0.074*	
C4	0.19150 (13)	0.53670 (14)	0.83396 (13)	0.0659 (6)	
H4	0.2238	0.5436	0.8887	0.079*	
C5	0.21785 (11)	0.51384 (12)	0.78609 (12)	0.0533 (5)	
H5	0.2680	0.5051	0.8092	0.064*	
C6	0.17118 (9)	0.50360 (10)	0.70431 (11)	0.0391 (4)	
C7	0.19871 (9)	0.47856 (9)	0.65004 (10)	0.0376 (4)	
H7	0.1727	0.4323	0.6237	0.045*	
C8	0.28056 (9)	0.46165 (10)	0.69505 (11)	0.0399 (4)	
C9	0.33020 (10)	0.52005 (11)	0.72284 (12)	0.0473 (5)	
C10	0.40660 (11)	0.50776 (14)	0.76622 (13)	0.0600 (6)	
H10	0.4390	0.5479	0.7834	0.072*	
C11	0.43294 (11)	0.43680 (15)	0.78296 (13)	0.0641 (6)	
H11	0.4836	0.4290	0.8120	0.077*	
C12	0.38526 (12)	0.37479 (13)	0.75730 (12)	0.0552 (5)	
C13	0.30753 (10)	0.38715 (11)	0.71243 (10)	0.0431 (4)	
C14	0.26164 (12)	0.32330 (11)	0.68782 (12)	0.0531 (5)	
H14	0.2108	0.3296	0.6590	0.064*	
C15	0.28989 (16)	0.25316 (13)	0.70521 (14)	0.0739 (7)	
H15	0.2584	0.2123	0.6876	0.089*	
C16	0.36600 (18)	0.24206 (16)	0.74933 (16)	0.0850 (9)	
H16	0.3850	0.1939	0.7615	0.102*	
C17	0.41189 (15)	0.30083 (17)	0.77423 (14)	0.0747 (8)	
H17	0.4624	0.2926	0.8033	0.090*	
C18	0.13247 (10)	0.52537 (10)	0.50919 (11)	0.0398 (4)	
C19	0.12036 (14)	0.59206 (12)	0.45734 (14)	0.0709 (7)	
H19A	0.1426	0.5835	0.4261	0.106*	
H19B	0.1422	0.6352	0.4906	0.106*	
H19C	0.0685	0.6002	0.4221	0.106*	
O1A	−0.04220 (8)	0.82226 (9)	0.16047 (8)	0.0572 (4)	
H1A1	0.0018	0.8190	0.1765	0.086*	
O2A	0.05897 (8)	0.65781 (7)	0.02450 (8)	0.0532 (4)	
H2A1	0.0688	0.6224	0.0051	0.080*	
O3A	0.11589 (7)	0.79276 (7)	0.26185 (8)	0.0472 (3)	
N1A	0.06323 (8)	0.72763 (8)	0.14574 (8)	0.0364 (3)	
H1A2	0.0553	0.6835	0.1257	0.044*	
C1A	−0.08229 (10)	0.80834 (10)	0.08044 (12)	0.0444 (4)	
C2A	−0.15812 (11)	0.80967 (13)	0.03873 (14)	0.0591 (6)	
H2A2	−0.1826	0.8196	0.0656	0.071*	
C3A	−0.19743 (13)	0.79645 (16)	−0.04221 (16)	0.0768 (8)	
H3A	−0.2486	0.7973	−0.0700	0.092*	
C4A	−0.16199 (13)	0.78190 (18)	−0.08270 (15)	0.0820 (8)	
H4A	−0.1888	0.7730	−0.1377	0.098*	
C5A	−0.08569 (12)	0.78056 (14)	−0.04061 (12)	0.0613 (6)	
H5A	−0.0617	0.7708	−0.0680	0.074*	
C6A	−0.04459 (10)	0.79339 (10)	0.04089 (11)	0.0412 (4)	
C7A	0.03910 (9)	0.79041 (9)	0.08923 (10)	0.0361 (4)	
H7A	0.0562	0.8361	0.1214	0.043*	
C8A	0.07419 (9)	0.78832 (9)	0.03854 (10)	0.0361 (4)	
C9A	0.08438 (10)	0.72246 (10)	0.00967 (11)	0.0421 (4)	
C10A	0.11902 (12)	0.72005 (12)	−0.03502 (12)	0.0547 (5)	
H10A	0.1268	0.6744	−0.0520	0.066*	
C11A	0.14087 (12)	0.78344 (13)	−0.05326 (12)	0.0573 (6)	
H11A	0.1644	0.7810	−0.0821	0.069*	
C12A	0.12871 (10)	0.85341 (11)	−0.02947 (11)	0.0471 (5)	
C13A	0.09502 (9)	0.85622 (10)	0.01715 (10)	0.0385 (4)	
C14A	0.08239 (10)	0.92775 (10)	0.03835 (12)	0.0478 (5)	
H14A	0.0605	0.9315	0.0689	0.057*	
C15A	0.10157 (12)	0.99152 (12)	0.01502 (14)	0.0618 (6)	
H15A	0.0927	1.0378	0.0299	0.074*	
C16A	0.13439 (13)	0.98752 (14)	−0.03111 (15)	0.0678 (7)	
H16A	0.1471	1.0310	−0.0471	0.081*	
C17A	0.14749 (12)	0.92046 (14)	−0.05222 (13)	0.0609 (6)	
H17A	0.1695	0.9183	−0.0826	0.073*	
C18A	0.09592 (9)	0.73285 (9)	0.22438 (10)	0.0376 (4)	
C19A	0.10774 (14)	0.66185 (12)	0.26910 (13)	0.0664 (6)	
H19D	0.1576	0.6455	0.2912	0.100*	
H19E	0.0746	0.6245	0.2332	0.100*	
H19F	0.0986	0.6698	0.3118	0.100*	

### Atomic displacement parameters (Å^2^)

**Table:**

	*U*^11^	*U*^22^	*U*^33^	*U*^12^	*U*^13^	*U*^23^
O1	0.0317 (7)	0.0809 (10)	0.0451 (8)	0.0032 (6)	0.0138 (6)	−0.0079 (7)
O2	0.0455 (8)	0.0528 (9)	0.0865 (11)	−0.0171 (6)	0.0263 (8)	−0.0101 (8)
O3	0.0459 (7)	0.0437 (7)	0.0373 (7)	−0.0037 (5)	0.0210 (6)	0.0021 (5)
N1	0.0413 (8)	0.0384 (8)	0.0412 (9)	−0.0073 (6)	0.0195 (7)	0.0029 (6)
C1	0.0359 (10)	0.0454 (10)	0.0392 (10)	−0.0024 (7)	0.0170 (8)	−0.0011 (8)
C2	0.0417 (11)	0.0628 (13)	0.0575 (13)	0.0017 (9)	0.0278 (10)	−0.0055 (10)
C3	0.0575 (14)	0.0827 (16)	0.0557 (14)	−0.0054 (11)	0.0367 (12)	−0.0149 (11)
C4	0.0558 (14)	0.0982 (18)	0.0403 (12)	−0.0084 (12)	0.0226 (11)	−0.0121 (11)
C5	0.0383 (10)	0.0776 (14)	0.0405 (12)	−0.0030 (9)	0.0181 (9)	−0.0024 (10)
C6	0.0347 (9)	0.0431 (9)	0.0384 (10)	−0.0037 (7)	0.0183 (8)	0.0017 (8)
C7	0.0344 (9)	0.0394 (9)	0.0353 (10)	−0.0045 (7)	0.0156 (8)	0.0017 (7)
C8	0.0336 (9)	0.0510 (10)	0.0345 (10)	−0.0024 (7)	0.0173 (8)	−0.0013 (8)
C9	0.0376 (10)	0.0579 (12)	0.0466 (12)	−0.0060 (8)	0.0221 (9)	−0.0049 (9)
C10	0.0359 (11)	0.0898 (17)	0.0515 (13)	−0.0125 (11)	0.0208 (10)	−0.0118 (11)
C11	0.0339 (11)	0.108 (2)	0.0452 (13)	0.0087 (11)	0.0170 (9)	−0.0039 (12)
C12	0.0496 (12)	0.0800 (15)	0.0343 (11)	0.0183 (11)	0.0207 (9)	0.0040 (10)
C13	0.0440 (10)	0.0565 (11)	0.0288 (9)	0.0069 (8)	0.0190 (8)	0.0016 (8)
C14	0.0631 (13)	0.0502 (11)	0.0416 (11)	0.0043 (9)	0.0243 (10)	0.0037 (9)
C15	0.103 (2)	0.0525 (13)	0.0569 (15)	0.0120 (12)	0.0349 (14)	0.0057 (11)
C16	0.116 (2)	0.0699 (17)	0.0615 (16)	0.0430 (17)	0.0412 (17)	0.0142 (14)
C17	0.0735 (16)	0.096 (2)	0.0478 (14)	0.0407 (15)	0.0266 (12)	0.0112 (13)
C18	0.0392 (10)	0.0421 (10)	0.0400 (11)	0.0004 (7)	0.0220 (8)	0.0062 (8)
C19	0.0904 (17)	0.0539 (13)	0.0561 (14)	−0.0071 (11)	0.0296 (13)	0.0165 (11)
O1A	0.0482 (8)	0.0822 (10)	0.0491 (9)	0.0035 (7)	0.0308 (7)	−0.0040 (7)
O2A	0.0785 (10)	0.0377 (7)	0.0558 (9)	−0.0008 (6)	0.0437 (8)	−0.0059 (6)
O3A	0.0497 (8)	0.0510 (8)	0.0430 (8)	−0.0150 (6)	0.0257 (6)	−0.0112 (6)
N1A	0.0441 (8)	0.0343 (7)	0.0327 (8)	0.0001 (6)	0.0212 (7)	−0.0004 (6)
C1A	0.0443 (11)	0.0470 (10)	0.0448 (11)	0.0062 (8)	0.0253 (9)	0.0098 (8)
C2A	0.0468 (12)	0.0752 (15)	0.0633 (15)	0.0115 (10)	0.0343 (11)	0.0171 (11)
C3A	0.0406 (12)	0.117 (2)	0.0626 (16)	0.0098 (12)	0.0201 (12)	0.0232 (14)
C4A	0.0492 (14)	0.140 (3)	0.0415 (13)	0.0071 (14)	0.0133 (11)	0.0125 (14)
C5A	0.0530 (13)	0.0939 (17)	0.0376 (12)	0.0075 (11)	0.0242 (10)	0.0092 (11)
C6A	0.0427 (10)	0.0449 (10)	0.0398 (10)	0.0053 (7)	0.0241 (8)	0.0085 (8)
C7A	0.0433 (10)	0.0335 (8)	0.0362 (10)	0.0019 (7)	0.0241 (8)	0.0024 (7)
C8A	0.0378 (9)	0.0396 (9)	0.0314 (9)	0.0033 (7)	0.0184 (8)	0.0018 (7)
C9A	0.0482 (11)	0.0436 (10)	0.0361 (10)	0.0038 (8)	0.0231 (9)	0.0012 (8)
C10A	0.0682 (14)	0.0587 (13)	0.0486 (12)	0.0087 (10)	0.0384 (11)	−0.0051 (10)
C11A	0.0640 (14)	0.0759 (15)	0.0486 (13)	0.0025 (11)	0.0410 (11)	−0.0010 (10)
C12A	0.0430 (11)	0.0617 (12)	0.0361 (10)	−0.0028 (8)	0.0203 (9)	0.0056 (9)
C13A	0.0350 (9)	0.0464 (10)	0.0310 (9)	−0.0001 (7)	0.0151 (8)	0.0038 (7)
C14A	0.0511 (11)	0.0443 (10)	0.0497 (12)	0.0021 (8)	0.0275 (10)	0.0048 (9)
C15A	0.0647 (14)	0.0450 (11)	0.0678 (15)	−0.0017 (9)	0.0291 (12)	0.0083 (10)
C16A	0.0716 (15)	0.0637 (15)	0.0650 (15)	−0.0153 (11)	0.0335 (13)	0.0165 (12)
C17A	0.0608 (13)	0.0790 (16)	0.0479 (13)	−0.0136 (11)	0.0319 (11)	0.0091 (11)
C18A	0.0360 (9)	0.0429 (10)	0.0353 (10)	−0.0027 (7)	0.0194 (8)	−0.0010 (8)
C19A	0.0892 (17)	0.0554 (13)	0.0411 (12)	−0.0012 (11)	0.0245 (12)	0.0092 (10)

### Geometric parameters (Å, °)

**Table:**

O1—C1	1.365 (2)	O1A—H1A1	0.8200
O1—H1	0.8200	O2A—C9A	1.372 (2)
O2—C9	1.361 (2)	O2A—H2A1	0.8200
O2—H2	0.8200	O3A—C18A	1.243 (2)
O3—C18	1.246 (2)	N1A—C18A	1.318 (2)
N1—C18	1.317 (2)	N1A—C7A	1.469 (2)
N1—C7	1.473 (2)	N1A—H1A2	0.8600
N1—H1A	0.8600	C1A—C2A	1.378 (3)
C1—C2	1.379 (3)	C1A—C6A	1.400 (3)
C1—C6	1.391 (2)	C2A—C3A	1.369 (3)
C2—C3	1.374 (3)	C2A—H2A2	0.9300
C2—H2A	0.9300	C3A—C4A	1.373 (4)
C3—C4	1.369 (3)	C3A—H3A	0.9300
C3—H3	0.9300	C4A—C5A	1.386 (3)
C4—C5	1.380 (3)	C4A—H4A	0.9300
C4—H4	0.9300	C5A—C6A	1.378 (3)
C5—C6	1.384 (3)	C5A—H5A	0.9300
C5—H5	0.9300	C6A—C7A	1.521 (2)
C6—C7	1.521 (3)	C7A—C8A	1.520 (2)
C7—C8	1.518 (2)	C7A—H7A	0.9800
C7—H7	0.9800	C8A—C9A	1.374 (2)
C8—C9	1.382 (3)	C8A—C13A	1.430 (2)
C8—C13	1.423 (3)	C9A—C10A	1.406 (3)
C9—C10	1.405 (3)	C10A—C11A	1.344 (3)
C10—C11	1.360 (3)	C10A—H10A	0.9300
C10—H10	0.9300	C11A—C12A	1.408 (3)
C11—C12	1.410 (3)	C11A—H11A	0.9300
C11—H11	0.9300	C12A—C17A	1.410 (3)
C12—C17	1.412 (3)	C12A—C13A	1.423 (3)
C12—C13	1.429 (3)	C13A—C14A	1.416 (3)
C13—C14	1.416 (3)	C14A—C15A	1.370 (3)
C14—C15	1.358 (3)	C14A—H14A	0.9300
C14—H14	0.9300	C15A—C16A	1.400 (4)
C15—C16	1.397 (4)	C15A—H15A	0.9300
C15—H15	0.9300	C16A—C17A	1.347 (3)
C16—C17	1.344 (4)	C16A—H16A	0.9300
C16—H16	0.9300	C17A—H17A	0.9300
C17—H17	0.9300	C18A—O3A	1.243 (2)
C18—O3	1.246 (2)	C18A—O3A	1.243 (2)
C18—C19	1.499 (3)	C18A—C19A	1.489 (3)
C19—H19A	0.9600	C19A—H19D	0.9600
C19—H19B	0.9600	C19A—H19E	0.9600
C19—H19C	0.9600	C19A—H19F	0.9600
O1A—C1A	1.357 (2)		
			
C1—O1—H1	109.5	C9A—O2A—H2A1	109.5
C9—O2—H2	109.5	C18A—N1A—C7A	125.89 (14)
C18—N1—C7	126.49 (14)	C18A—N1A—H1A2	117.1
C18—N1—H1A	116.8	C7A—N1A—H1A2	117.1
C7—N1—H1A	116.8	O1A—C1A—C2A	121.13 (18)
O1—C1—C2	122.04 (16)	O1A—C1A—C6A	118.25 (16)
O1—C1—C6	116.91 (17)	C2A—C1A—C6A	120.61 (19)
C2—C1—C6	121.03 (17)	C3A—C2A—C1A	120.1 (2)
C3—C2—C1	120.10 (18)	C3A—C2A—H2A2	119.9
C3—C2—H2A	120.0	C1A—C2A—H2A2	119.9
C1—C2—H2A	120.0	C2A—C3A—C4A	120.6 (2)
C4—C3—C2	119.9 (2)	C2A—C3A—H3A	119.7
C4—C3—H3	120.1	C4A—C3A—H3A	119.7
C2—C3—H3	120.1	C3A—C4A—C5A	119.2 (2)
C3—C4—C5	120.1 (2)	C3A—C4A—H4A	120.4
C3—C4—H4	120.0	C5A—C4A—H4A	120.4
C5—C4—H4	120.0	C6A—C5A—C4A	121.6 (2)
C6—C5—C4	121.34 (19)	C6A—C5A—H5A	119.2
C6—C5—H5	119.3	C4A—C5A—H5A	119.2
C4—C5—H5	119.3	C5A—C6A—C1A	117.88 (17)
C5—C6—C1	117.60 (18)	C5A—C6A—C7A	122.95 (17)
C5—C6—C7	122.49 (16)	C1A—C6A—C7A	119.16 (16)
C1—C6—C7	119.90 (16)	N1A—C7A—C8A	111.85 (13)
N1—C7—C8	110.73 (14)	N1A—C7A—C6A	109.32 (13)
N1—C7—C6	110.26 (14)	C8A—C7A—C6A	114.26 (14)
C8—C7—C6	113.48 (14)	N1A—C7A—H7A	107.0
N1—C7—H7	107.4	C8A—C7A—H7A	107.0
C8—C7—H7	107.4	C6A—C7A—H7A	107.0
C6—C7—H7	107.4	C9A—C8A—C13A	118.19 (16)
C9—C8—C13	119.13 (17)	C9A—C8A—C7A	121.65 (15)
C9—C8—C7	119.21 (16)	C13A—C8A—C7A	120.11 (15)
C13—C8—C7	121.61 (15)	O2A—C9A—C8A	118.40 (16)
O2—C9—C8	117.37 (16)	O2A—C9A—C10A	119.78 (16)
O2—C9—C10	120.90 (18)	C8A—C9A—C10A	121.81 (17)
C8—C9—C10	121.71 (19)	C11A—C10A—C9A	120.26 (18)
C11—C10—C9	119.6 (2)	C11A—C10A—H10A	119.9
C11—C10—H10	120.2	C9A—C10A—H10A	119.9
C9—C10—H10	120.2	C10A—C11A—C12A	121.17 (19)
C10—C11—C12	121.49 (19)	C10A—C11A—H11A	119.4
C10—C11—H11	119.3	C12A—C11A—H11A	119.4
C12—C11—H11	119.3	C11A—C12A—C17A	121.7 (2)
C11—C12—C17	122.1 (2)	C11A—C12A—C13A	118.89 (17)
C11—C12—C13	118.98 (19)	C17A—C12A—C13A	119.44 (19)
C17—C12—C13	119.0 (2)	C14A—C13A—C12A	117.06 (16)
C14—C13—C8	123.81 (17)	C14A—C13A—C8A	123.40 (17)
C14—C13—C12	117.12 (18)	C12A—C13A—C8A	119.53 (16)
C8—C13—C12	119.06 (18)	C15A—C14A—C13A	121.6 (2)
C15—C14—C13	121.8 (2)	C15A—C14A—H14A	119.2
C15—C14—H14	119.1	C13A—C14A—H14A	119.2
C13—C14—H14	119.1	C14A—C15A—C16A	120.4 (2)
C14—C15—C16	120.4 (3)	C14A—C15A—H15A	119.8
C14—C15—H15	119.8	C16A—C15A—H15A	119.8
C16—C15—H15	119.8	C17A—C16A—C15A	119.7 (2)
C17—C16—C15	120.1 (2)	C17A—C16A—H16A	120.2
C17—C16—H16	119.9	C15A—C16A—H16A	120.2
C15—C16—H16	119.9	C16A—C17A—C12A	121.8 (2)
C16—C17—C12	121.6 (2)	C16A—C17A—H17A	119.1
C16—C17—H17	119.2	C12A—C17A—H17A	119.1
C12—C17—H17	119.2	O3A—C18A—N1A	123.91 (16)
O3—C18—N1	124.33 (16)	O3A—C18A—N1A	123.91 (16)
O3—C18—N1	124.33 (16)	O3A—C18A—N1A	123.91 (16)
O3—C18—C19	119.64 (17)	O3A—C18A—C19A	119.49 (17)
O3—C18—C19	119.64 (17)	O3A—C18A—C19A	119.49 (17)
N1—C18—C19	116.02 (17)	O3A—C18A—C19A	119.49 (17)
C18—C19—H19A	109.5	N1A—C18A—C19A	116.59 (16)
C18—C19—H19B	109.5	C18A—C19A—H19D	109.5
H19A—C19—H19B	109.5	C18A—C19A—H19E	109.5
C18—C19—H19C	109.5	H19D—C19A—H19E	109.5
H19A—C19—H19C	109.5	C18A—C19A—H19F	109.5
H19B—C19—H19C	109.5	H19D—C19A—H19F	109.5
C1A—O1A—H1A1	109.5	H19E—C19A—H19F	109.5
			
O1—C1—C2—C3	177.50 (19)	C6A—C1A—C2A—C3A	−0.1 (3)
C6—C1—C2—C3	−1.1 (3)	C1A—C2A—C3A—C4A	−0.1 (4)
C1—C2—C3—C4	0.1 (3)	C2A—C3A—C4A—C5A	0.1 (4)
C2—C3—C4—C5	0.2 (4)	C3A—C4A—C5A—C6A	0.1 (4)
C3—C4—C5—C6	0.4 (4)	C4A—C5A—C6A—C1A	−0.3 (3)
C4—C5—C6—C1	−1.3 (3)	C4A—C5A—C6A—C7A	178.4 (2)
C4—C5—C6—C7	179.9 (2)	O1A—C1A—C6A—C5A	−178.92 (18)
O1—C1—C6—C5	−177.01 (17)	C2A—C1A—C6A—C5A	0.3 (3)
C2—C1—C6—C5	1.7 (3)	O1A—C1A—C6A—C7A	2.3 (2)
O1—C1—C6—C7	1.8 (2)	C2A—C1A—C6A—C7A	−178.44 (17)
C2—C1—C6—C7	−179.54 (17)	C18A—N1A—C7A—C8A	122.33 (17)
C18—N1—C7—C8	120.86 (18)	C18A—N1A—C7A—C6A	−110.10 (18)
C18—N1—C7—C6	−112.71 (19)	C5A—C6A—C7A—N1A	−114.0 (2)
C5—C6—C7—N1	−121.32 (19)	C1A—C6A—C7A—N1A	64.7 (2)
C1—C6—C7—N1	59.9 (2)	C5A—C6A—C7A—C8A	12.2 (2)
C5—C6—C7—C8	3.5 (2)	C1A—C6A—C7A—C8A	−169.11 (15)
C1—C6—C7—C8	−175.20 (15)	N1A—C7A—C8A—C9A	39.0 (2)
N1—C7—C8—C9	49.0 (2)	C6A—C7A—C8A—C9A	−85.9 (2)
C6—C7—C8—C9	−75.7 (2)	N1A—C7A—C8A—C13A	−143.40 (15)
N1—C7—C8—C13	−133.86 (17)	C6A—C7A—C8A—C13A	91.73 (19)
C6—C7—C8—C13	101.53 (19)	C13A—C8A—C9A—O2A	−174.72 (15)
C13—C8—C9—O2	−176.66 (17)	C7A—C8A—C9A—O2A	2.9 (3)
C7—C8—C9—O2	0.6 (3)	C13A—C8A—C9A—C10A	4.5 (3)
C13—C8—C9—C10	1.5 (3)	C7A—C8A—C9A—C10A	−177.87 (17)
C7—C8—C9—C10	178.75 (18)	O2A—C9A—C10A—C11A	176.72 (19)
O2—C9—C10—C11	176.8 (2)	C8A—C9A—C10A—C11A	−2.5 (3)
C8—C9—C10—C11	−1.3 (3)	C9A—C10A—C11A—C12A	−1.0 (3)
C9—C10—C11—C12	0.4 (3)	C10A—C11A—C12A—C17A	−176.2 (2)
C10—C11—C12—C17	179.9 (2)	C10A—C11A—C12A—C13A	2.3 (3)
C10—C11—C12—C13	0.2 (3)	C11A—C12A—C13A—C14A	−178.70 (18)
C9—C8—C13—C14	179.16 (18)	C17A—C12A—C13A—C14A	−0.2 (3)
C7—C8—C13—C14	2.0 (3)	C11A—C12A—C13A—C8A	−0.2 (3)
C9—C8—C13—C12	−0.8 (3)	C17A—C12A—C13A—C8A	178.39 (17)
C7—C8—C13—C12	−177.97 (17)	C9A—C8A—C13A—C14A	175.33 (17)
C11—C12—C13—C14	179.99 (19)	C7A—C8A—C13A—C14A	−2.4 (3)
C17—C12—C13—C14	0.3 (3)	C9A—C8A—C13A—C12A	−3.1 (2)
C11—C12—C13—C8	−0.1 (3)	C7A—C8A—C13A—C12A	179.18 (15)
C17—C12—C13—C8	−179.79 (19)	C12A—C13A—C14A—C15A	0.1 (3)
C8—C13—C14—C15	179.4 (2)	C8A—C13A—C14A—C15A	−178.39 (18)
C12—C13—C14—C15	−0.7 (3)	C13A—C14A—C15A—C16A	0.2 (3)
C13—C14—C15—C16	0.9 (4)	C14A—C15A—C16A—C17A	−0.4 (3)
C14—C15—C16—C17	−0.7 (4)	C15A—C16A—C17A—C12A	0.3 (4)
C15—C16—C17—C12	0.3 (4)	C11A—C12A—C17A—C16A	178.5 (2)
C11—C12—C17—C16	−179.8 (2)	C13A—C12A—C17A—C16A	−0.1 (3)
C13—C12—C17—C16	−0.1 (4)	C7A—N1A—C18A—O3A	−5.8 (3)
C7—N1—C18—O3	−2.7 (3)	C7A—N1A—C18A—O3A	−5.8 (3)
C7—N1—C18—O3	−2.7 (3)	C7A—N1A—C18A—O3A	−5.8 (3)
C7—N1—C18—C19	177.57 (19)	C7A—N1A—C18A—C19A	173.15 (18)
O1A—C1A—C2A—C3A	179.1 (2)		

### Hydrogen-bond geometry (Å, °)

**Table:**

*D*—H···*A*	*D*—H	H···*A*	*D*···*A*	*D*—H···*A*
O1—H1···O3^i^	0.82	1.99	2.792 (2)	166
O2A—H2A1···O3^ii^	0.82	1.89	2.702 (2)	170
O2—H2···O3A^iii^	0.82	1.81	2.588 (2)	159
O1A—H1A1···O3A	0.82	2.16	2.928 (2)	156
C7—H7···O3	0.98	2.51	2.901 (2)	104
C7A—H7A···O3A	0.98	2.47	2.879 (2)	105

Symmetry codes: (i) −*x*, −*y*+1, −*z*+1; (ii) *x*, −*y*+1, *z*−1/2; (iii) −*x*+1/2, −*y*+3/2, −*z*+1.
